# HOXA-AS2 may be a potential prognostic biomarker in human cancers: A meta-analysis and bioinformatics analysis

**DOI:** 10.3389/fgene.2022.944278

**Published:** 2022-11-10

**Authors:** Fan Zhang, Guangming Zhang, Helin Zhang, Xingyu Pu, Fei Chi, Dengxiao Zhang, Xiaoming Xin, Mingxuan Gao, Wenyuan Luo, Xingyong Li

**Affiliations:** ^1^ The First Clinical Medical College of Gansu University of Chinese Medicine (Gansu Provincial Hospital), Lanzhou, China; ^2^ Department of Orthopedics, Gansu Provincial Hospital, Lanzhou, China

**Keywords:** lncRNA, HOXA-AS2, cancers, prognosis, meta-analysis, bioinformatics analysis

## Abstract

**Background:** Dysregulation of long non-coding (lncRNA) has been reported in various solid tumors. HOXA cluster antisense RNA 2 (HOXA-AS2) is a newly identified lncRNA with abnormal expression in several human malignancies. However, its prognostic value remains controversial. This meta-analysis synthesized available data to clarify the association between HOXA-AS2 expression levels and clinical prognosis in multiple cancers.

**Methods:** Four public databases (Embase, PubMed, Web of Science, The Cochrane Library) were used to identify eligible studies. Hazard ratios (HRs) and odds ratios (ORs) with their 95% confidence intervals (CIs) were combined to assess the correlation of HOXA-AS2 expression with survival outcomes and clinicopathological features of cancer patients. Publication bias was measured using Begg’s funnel plot and Egger’s regression test, and the stability of the combined results was measured using sensitivity analysis. Additionally, multiple public databases were screened and extracted to validate the results of this meta-analysis.

**Results:** The study included 20 studies, containing 1331 patients. The meta-analysis showed that the overexpression of HOXA-AS2 was associated with poor overall survival (HR = 2.06, 95% CI 1.58–2.69, *p* < 0.001). In addition, the high expression of HOXA-AS2 could forecast advanced tumor stage (OR = 3.89, 95% CI 2.90–5.21, *p* < 0.001), earlier lymph node metastasis (OR = 3.48, 95% CI 2.29–5.29, *p* < 0.001), larger tumor size (OR = 2.36, 95% CI 1.52–3.66, *p* < 0.001) and earlier distant metastasis (OR = 3.54, 95% CI 2.00–6.28, *p* < 0.001). However, other clinicopathological features, including age (OR = 1.09, 95% CI 0.86–1.38, *p* = 0.467), gender (OR = 0.92, 95% CI 0.72–1.18, *p* = 0.496), depth of invasion (OR = 2.13, 95% CI 0.77–5.90, *p* = 0.146) and differentiation (OR = 1.02, 95% CI 0.65–1.59, *p* = 0.945) were not significantly different from HOXA-AS2 expression.

**Conclusion:** Our study showed that the overexpression of HOXA-AS2 was related to poor overall survival and clinicopathological features. HOXA-AS2 may serve as a potential prognostic indicator and therapeutic target for tumor treatment.

## Introduction

Cancer is the second greatest cause of death in most parts of the world, and it has become the most common public pathological condition on the planet (Chu et al., 2020). Traditional cancer treatments, such as surgery, adjuvant medical treatment, and actinotherapy have improved dramatically over the last century (Zhang et al., 2019). Despite this, 5-year cancer survival rates remain poor, particularly for patients with advanced tumor stage or metastasis (Li et al., 2019). One of the most significant causes is the lack of a good biomarker for detecting cancer early and predicting the clinical outcome of cancer patients (Ye et al., 2019). The significance of biomarkers in cancer has garnered increased attention in recent years across various fields, and they are thought to play critical roles in effectively screening or diagnosing cancer (Tang et al., 2020).

Long noncoding RNAs (lncRNAs) are RNA molecules that are longer than 200 nucleotides and cannot code for proteins (Zhou et al., 2019). A huge number of lncRNAs are produced during the active transcription of the human genome (Morlando and Fatica, 2018). One of the functions of lncRNAs *in vivo* is as tumor suppressors or oncogenes (Xu et al., 2021). Increasing evidence suggest that lncRNAs play a synergistic role in tumorigenesis or tumor suppression and that aberrant lncRNA expression is linked to cell proliferation, growth, and metastasis (Wang Y. et al., 2020). The development of RNA-targeted therapies has presented possibility of lncRNA-guided cancer therapy (Bhan et al., 2017). The inhibition of lncRNA function by RNA depletion and the removal of lncRNA exons encoding essential functional domains using splice-switching oligonucleotides may be the mechanism for targeting lncRNAs for cancer therapy (Kole et al., 2012; Schmitt and Chang, 2016). Therefore, functional lncRNA can be used as a biomarker for cancer diagnosis and for predicting treatment outcome and patient prognosis (Wang J. et al., 2020).

LncRNA HOXA cluster antisense RNA 2 (HOXA-AS2) is located on chromosome 7p15.2, a 1048-bp lncRNA, between the HOXA3 and HOXA4 genes of the HOXA cluster (Liu et al., 2019). Previous studies found that HOXA-AS2 was up-regulated in certain cancers. The increased expression of HOXA-AS2 typically predicts poor prognosis for patients with several cancers including cervical cancer (CC) (Chen and He, 2021), oral squamous cell carcinoma (OSCC) (Chen et al., 2021), lung cancer (LC) (Li and Jiang, 2017; Cui et al., 2019; Liu et al., 2019), colorectal cancer (CRC) (Li et al., 2016; Ding et al., 2017), breast cancer (BC) (Fang et al., 2017), thyroid cancer (TC) (Xia et al., 2018; Jiang et al., 2019), hepatocellular carcinoma (HCC) (Wang et al., 2016; Zhang et al., 2018; Lu et al., 2020), acute myeloid leukemia (AML) (Qu et al., 2020), bladder cancer (Wang F. et al., 2019), osteosarcoma (OSA) (Wang et al., 2018; Wang L. et al., 2019), lower-grade glioma (LGG) (Wu et al., 2019), prostate cancer (PCa) (Xiao and Song, 2020), gastric cancer (GC) (Xie et al., 2015). A high level of HOXA-AS2 expression is associated with poor overall survival (OS) and clinicopathological characteristics such as differentiation, tumor node metastasis (TNM) stage, lymph node metastasis (LNM). However, it is not clear the prognostic value of HOXA-AS2, as most of the published studies were based on a small group of patients. We explored the prognostic value of HOXA-AS2 in pan-cancer for the first time using meta-analysis. Furthermore, we further validated and explored the prognostic value of HOXA-AS2 in multiple databases through bioinformatics analysis, and explored HOXA-AS2-related genes and potential pathways. Also, the role of HOXA-AS2 in tumor immunity was investigated to identify the potential of HOXA-AS2 as a novel tumor marker and therapeutic target.

## Materials and methods

### Registration

The study was registered in the International Platform of Registered Systematic Review and Meta-Analysis Protocols (the registration number is: CRD42021292257). Because the present study was a systematic review and meta-analysis, Institutional Review Board (IRB) approval was not required.

### Search strategy

Quality meta-analysis guidelines were followed to search for and find related papers in the Embase, PubMed, Web of Science, and The Cochrane Library. Key terms include the following: “HOXA-AS2” “long noncoding RNA HOXA-AS2” “lncRNA HOXA-AS2” “HOXA cluster antisense RNA 2” “HOXA3as” “neoplasm” “cancer” “malignancy” “neoplasia” “melanoma” “tumor” “sarcoma” “carcinoma” or “adenoma”. These terms were used to maximize the likelihood of finding a relevant article. The literature search included articles revealed as of 15 November 2021. A manual search of the reference lists of the retrieved literature was performed to confirm the eligible studies included. Any conflicts between the inclusion and exclusion clauses were resolved through group discussion.

### Participants, interventions, and comparators

Studies that complied with the following criteria were eventually included: The inclusion criteria were: (a) the use of real-time quantitative polymerase chain reaction (RT-qPCR) analysis to determine the expression of HOXA-AS2 in neoplastic tissues; (b) patients diagnosed with cancer, and the study described a link between HOXA-AS2 and survival data or clinicopathology; (c) the patients were divided into two groups according to the expression level of HOXA-AS2, and (d) the quantitative hazard ratios (HRs) of OS could be extracted from the text or survival curve. The exclusion criteria were: (a) studies not related to tumors or HOXA-AS2; (b) duplicate publications; (c) reviews, conference abstracts, or case reports, and (d) studies that lacked relevant data.

### Data extraction

Two researchers extracted information from each study, and any disagreement was resolved by discussing it with a third author. We obtained the following data and information from every study: (a) first author, (b) publication year, (c) country of origin, (d) cancer type, (e) number of samples, (f) HOXA-AS2 expression detection technique, (g) cut-off value, (h) sample size with high and low HOXA-AS2 expression, (i) HRs and 95% confidence intervals (CIs) for OS, (j) clinicopathologic parameters, and (k) follow-up times. OS data was directly obtained or extracted from the Kaplan-Meier (KM) curves using Engauge Digitizer version 4.1 software and the HRs and 95% CIs were computed.

#### Quality assessment

Two reviewers extracted information individually based on the inclusion and exclusion criteria. Some disagreements were resolved in consultation with a third reviewer. The quality of the studies was assessed using the Newcastle–Ottawa scale (NOS). The scale uses nine elements to judge a study, and a score of one is satisfied for an exact item. Total scores range from 0 to 9. A NOS score of ≥ 7 represents high-quality analysis results.

### Statistical analysis

All statistical analyses were conducted using Stata software (version 12.0). The correlation of HOXA-AS2 expression with survival and clinicopathological features of tumor patients was assessed using HRs and odds ratios (ORs) with their 95% CIs, respectively. The chi-squared test and *I* ^*2*^ statistic were preferred to determine the heterogeneity between studies. If there is strong heterogeneity (*P*_*Q*_ < 0.1, *I* ^2^ > 50%), we considered the random-effect model was applied, and the fixed-effect model was applied otherwise. All results are shown as Forest plots. Egger’s test and Begg’s funnel plot were used to evaluate publication bias, and sensitivity analysis was conducted to evaluate the robustness of the results.

### Public data and tools

HOXA-AS2 expression levels in tumors and normal tissues of different solid tumors were analyzed by the Gene Expression Profiling Interactive Analysis (GEPIA, http://gepia.cancer-pku.cn) online database (based on TCGA and GTEx databases) (cutoff, *p* < 0.01). The survival outcomes were then verified by plotting the correlation between HOXA-AS2 expression and OS as a KM curve. Moreover, we further explored the prognostic value of HOXA-AS2 in various cancers using the Biomarker Exploration of Solid Tumors (BEST, https://rookieutopia.com) online tool. Additionally, we further explored the correlation between HOXA-AS2 and drug response through the CellMiner database (Reinhold et al., 2012) using the R-package “readxl”, “impute” and “limma” options.

### Correlation of HOXA-AS2 expression with tumor immunity

Initially, we analyzed the relationship between HOXA-AS2 expression and the level of immune cell infiltration (ICI) in various cancers based on the R packages “ggExtra”, “ggpubr” and “ggplot2” via the CIBERSORT tool. Next, stromal and immune scores were calculated for each tumor sample using the ESTIMATE algorithm. The correlation between HOXA-AS2 expression and tumor microenvironment (TME) was assessed by the R package “ESTIMATE” and “limma”. In addition, the relation of HOXA-AS2 expression and tumor mutational load (TMB), tumor microsatellite instability (MSI) and immune checkpoint genes were further evaluated. TMB scores were calculated by Perl scripts, and MSI scores were determined from TCGA database mutation data. The results were visualized using the R package “RColorBrewer” and “reshape2”.

### Analysis of HOXA-AS2-related genes and construction of signaling pathway network

To further investigate the value of HOXA-AS2, we obtained related genes from the MEM-Multi Experiment Matrix database. Gene Ontology (GO) and Kyoto Encyclopedia of Genes and Genomes (KEGG) analyses were carried out. The top findings with a *p*-value of less than 0.05 were deemed significant. Finally, we used Cytoscape software to create a signal pathway network.

## Results

### Identification of articles

**FIGURE 1:**
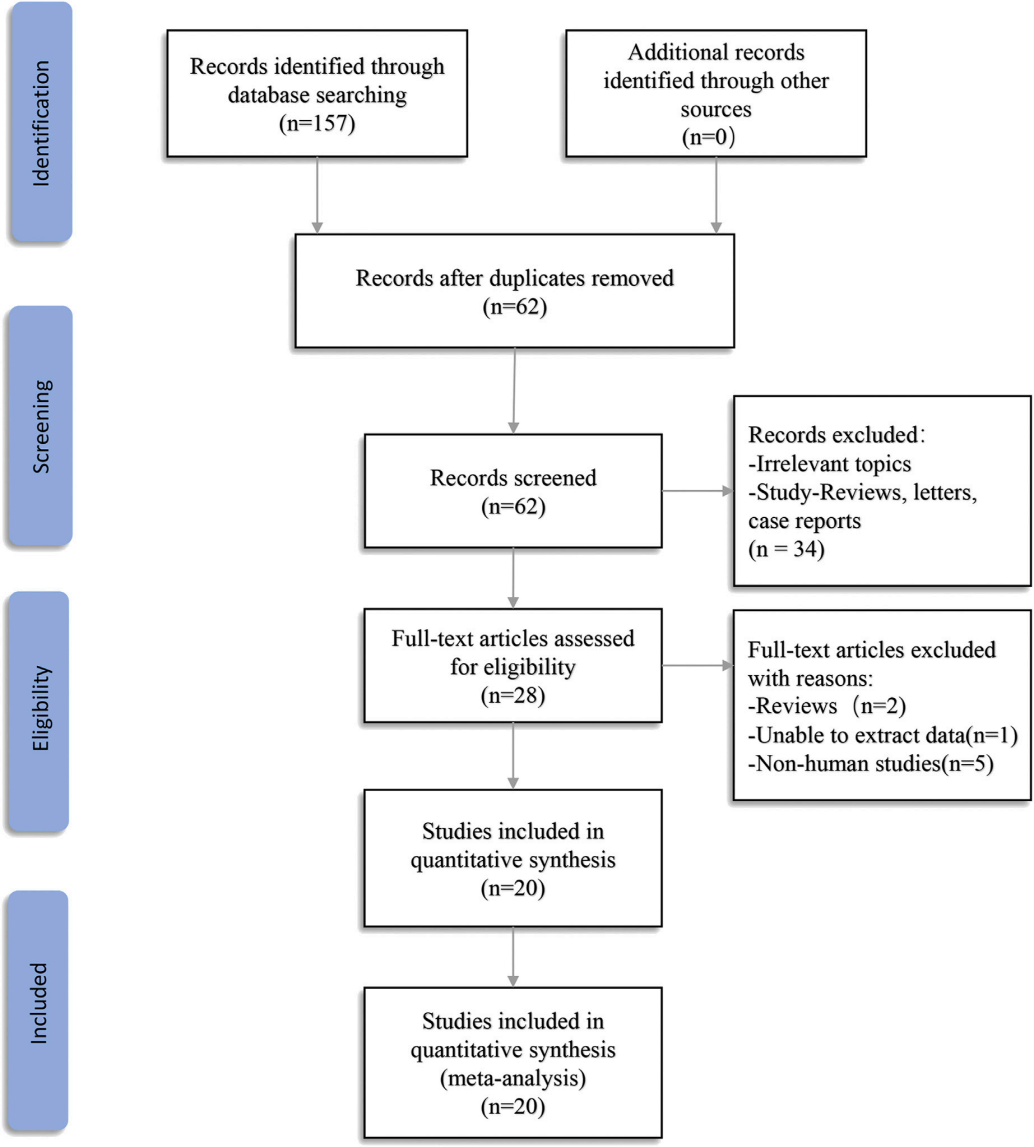
Flow diagram of this meta-analysis.

A total of 157 records were found in four electronic databases (Embase = 57, PubMed = 47, Web of Science = 53, The Cochrane Library = 0). Ninety-five duplicate articles were deleted using Endnote X9 software. After screening the titles and abstracts, 34 articles were excluded because they were not associated with the review topic or as a result of reviews, meta-analysis, letters, or expert opinions. Hence, a full-text examination was conducted for 28 articles. One article was excluded because we were unable to extract data. Two papers were eliminated because they were reviews, and five articles were eliminated because they were cell-based studies. In the end, 20 articles were included in the final meta-analysis (Figure 1).

### Characteristics of the included articles

All selected articles were published between 2015 and 2021 and included 1331 patients, all of whom were from China. The smallest sample size was 27, and the largest sample size was 128. Among the twenty studies, one focused on LGG (Wu et al., 2019), one on CC (Chen and He, 2021), one on OSCC (Chen et al., 2021), three on LC (Li and Jiang, 2017; Cui et al., 2019; Liu et al., 2019), two on CRC (Li et al., 2016; Ding et al., 2017), one on BC (Fang et al., 2017), two on TC (Xia et al., 2018; Jiang et al., 2019), three on HCC (Wang et al., 2016; Zhang et al., 2018; Lu et al., 2020), two on OSA (Wang et al., 2018; Wang L. et al., 2019), one on PCa (Xiao and Song, 2020), one on GC (Xie et al., 2015), one on bladder cancer (Wang F. et al., 2019), and one on AML (Qu et al., 2020). The expression of the indicated genes in cancer tissues was measured by RT-qPCR. All eligible studies were dichotomized into low and high HOXA-AS2 expression groups based on a cut-off value. The follow-up time ranged from 25 to 120 months. All included studies were cohort studies, 65% (13/20) of which reported OS rates. The main characteristics of the eligible studies are shown in Table 1.

**TABLE 1:** Characteristics of studies in this meta-analysis.

Study	Year	Country	Cancer type	Sample type	Total Size(n)	Detection Method	Cutoff	Outcome	Multivariate Analysis	HR statistic	NOS score
Chen RH	2021	China	CC	Tissue	27	RT-qPCR	mean	OS	NR	Rep	6
Chen RW	2021	China	OSCC	Tissue	46	RT-qPCR	NR	NR	NR	NR	5
Cui	2019	China	LC	Tissue	80	RT-qPCR	mean	OS	NR	SC	7
Ding	2017	China	CRC	Tissue	69	RT-qPCR	NR	NR	NR	NR	5
Fang	2017	China	BC	Tissue	38	RT-qPCR	NR	OS	NR	SC	6
Jiang	2019	China	TC	Tissue	68	NR	mean	OS	NR	SC	7
Li	2016	China	CRC	Tissue	30	RT-qPCR	NR	OS	NR	SC	6
Li	2017	China	LC	Tissue	103	RT-qPCR	median	OS	Yes	SC	8
Liu	2019	China	LC	Tissue	52	RT-qPCR	median	OS	NR	SC	7
Lu	2020	China	HCC	Tissue	106	RT-qPCR	median	OS	Yes	Rep	8
Qu	2020	China	AML	Blood	108	RT-qPCR	median	OS	Yes	Rep	8
Wang F	2016	China	HCC	Tissue	112	RT-qPCR	NR	OS	NR	SC	6
Wang Y	2018	China	OS	Tissue	66	RT-qPCR	NR	NR	NR	NR	5
Wang L	2019	China	OSA	Tissue	27	RT-qPCR	mean	OS	NR	SC	6
Wang F	2019	China	Bladder cancer	tissue	80	RT-qPCR	NR	NR	NR	NR	5
Wu	2019	China	LGG	tissue	50	RT-qPCR	NR	NR	NR	NR	5
Xia	2018	China	TC	tissue	128	RT-qPCR	mean	NR	NR	NR	6
Xiao	2020	China	PCa	tissue	68	RT-qPCR	mean	OS	NR	SC	7
Xie	2015	China	GC	tissue	55	RT-qPCR	median	OS	NR	SC	7
Zhang	2018	China	HCC	tissue	58	RT-qPCR	NR	NR	NR	NR	5

HR, hazard ratio; GC, gastric cancer; CRC, colorectal cancer; HCC, hepatocellular carcinoma; PCa, Prostate cancer; CC, cervical cancer; OSA, osteosarcoma; LGG, lower-grade glioma; TC, thyroid Cancer; AML, acute myeloid leukemia; OSCC, oral squamous cell carcinoma; LC, lung cancer; BC, breast cancer; NR, no report; OS, overall survival; PFS, progression-free survival; Rep, report; SC, survival curve; RT-qPCR, real-time quantitative polymerase chain reaction.

### Association of HOXA-AS2 with OS

**FIGURE 2:**
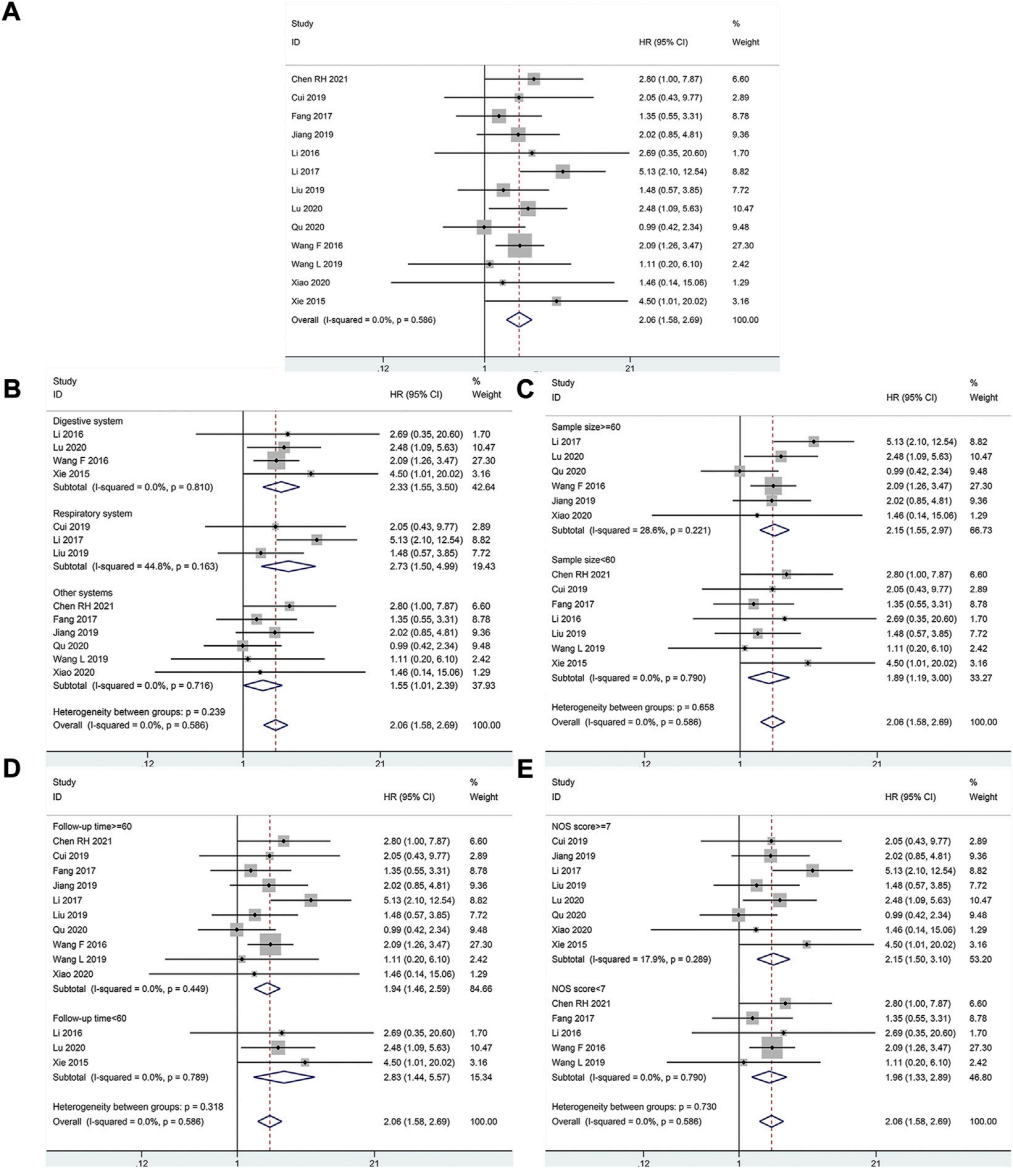
Relationship between HOXA-AS2 expression and overall survival. **(A)** Forest plots for association of HOXA-AS2 expression with overall survival. **(B)** Subgroup analysis stratified by cancer type. **(C)** Subgroup analysis stratified by sample size. **(D)** Subgroup analysis stratified by follow-up time. **(E)** Subgroup analysis stratified by NOS score.

A total of 834 patients in 13 studies reported a link between HOXA-AS2 expression and OS. Since there was no significant heterogeneity between the studies, a fixed-effects model was used to calculate the HR and 95% CI. The pooled HR was 2.06, which indicated that HOXA-AS2 overexpression predicted poor OS in these patients with neoplasms (Figure 2A). Furthermore, KM survival analysis was applied to determine OS in different subgroups of patients according to tumor type (digestive system, respiratory system, others) (Figure 2B), sample size (n ≥ 60 or n < 60) (Figure 2C), follow-up time (≥60 months or < 60 months) (Figure 2D), and NOS score (NOS scores ≥ 7 or < 7) (Figure 2E). As depicted in Table 2, higher HOXA-AS2 expression levels were significantly associated with worse OS.

**TABLE 2:** Subgroup meta-analysis of pooled HRs for OS.

Stratified analysis	Studies (n)	Number of patients	Pooled HR (95% CI)	*P*-value	Heterogeneity		
					*I* ^*2*^, %	*P*-value	Model
Cancer type							
Digestive system	4	303	2.33(1.55–3.50)	<0.001	0.0	0.810	Fixed
Respiratory system	3	195	2.73(1.50–4.99)	0.001	44.8	0.163	Fixed
Other systems	6	336	1.55(1.01–2.39)	0.046	0.0	0.716	Fixed
Sample size							
≥60	6	565	2.15(1.55–2.97)	<0.001	28.6	0.221	Fixed
<60	7	269	1.89(1.19–3.00)	0.007	0.0	0.790	Fixed
Follow-up time (month)							
≥60	10	643	1.94(1.46–2.59)	<0.001	0.0	0.449	Fixed
<60	3	191	2.83(1.44–5.57)	<0.001	0.0	0.789	Fixed
NOS score							
≥7	8	600	2.15(1.50–3.10)	<0.001	17.9	0.289	Fixed
<7	5	234	1.96(1.33–2.89)	0.001	20.6	0.790	Fixed

CI, Confidence interval; HR, Hazard ratio.

### Association between HOXA-AS2 and clinicopathologic parameters

**FIGURE 3:**
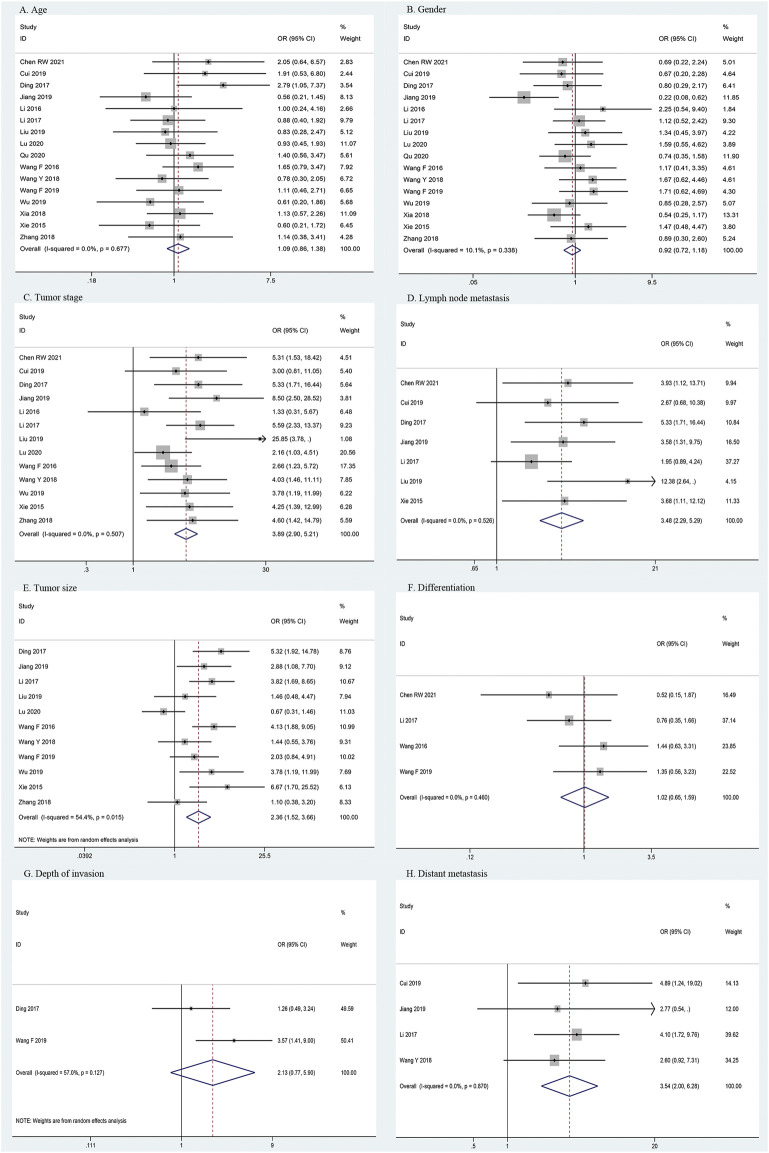
Forest plots for association of HOXA-AS2 expression with clinicopathological features. **(A)** Age. **(B)** Gender. **(C)** Tumor stage. **(D)** Lymph node metastasis. **(E)** Tumor size. **(F)** Differentiation. **(G)** Depth of invasion. **(H)** Distant metastasis.

Correlations between HOXA-AS2 expression and the clinicopathological features of the patients are shown in Table 3. The meta-analysis results showed that higher HOXA-AS2 expression levels tended to be significantly associated with advanced tumor stage (OR = 3.89, 95% CI 2.90–5.21, *p* < 0.001) (Figure 3C), earlier LNM (OR = 3.48, 95% CI 2.29–5.29, *p* < 0.001) (Figure 3D), larger tumor size (OR = 2.36, 95% CI 1.52–3.66, *p* < 0.001) (Figure 3E) and earlier distant metastasis (OR = 3.54, 95% CI 2.00–6.28, *p* < 0.001) (Figure 3H). However, age (OR = 1.09, 95% CI 0.86–1.38, *p* = 0.467) (Figure 3A), gender (OR = 0.92, 95% CI 0.72–1.18, *p* = 0.496) (Figure 3B), depth of invasion (OR = 2.13, 95% CI 0.77–5.90, *p* = 0.146) (Figure 3G), and differentiation (OR = 1.02, 95% CI 0.65–1.59, *p* = 0.945) (Figure 3F), had no significant link with increased HOXA-AS2 expression levels.

**TABLE 3:** Association of HOXA-AS2 expression with clinicopathological features.

Clinicopathological parameters	Patients (n)	Or (95%CI)	*P* Value	Heterogeneity (*I* ^*2*^, *P*)	Model
Age (elderly vs. nonelderly)	1181	1.09(0.86–1.38)	0.467	0.0%, 0.677	Fixed
Gender (male vs. female)	1185	0.92(0.72–1.18)	0.496	10.1%, 0.338	Fixed
Tumor stage (III + IV vs. I + II)	551	3.89(2.90–5.21)	<0.001	0.0%, 0.507	Fixed
Lymph node metastasis (positive vs. negative)	433	3.48(2.29–5.29)	<0.001	0.0%, 0.526	Fixed
Tumor size (big vs. small)	829	2.36(1.52–3.66)	<0.001	54.4%, 0.015	Random
Differentiation (poor vs. well)	341	1.02(0.65–1.59)	0.945	0.0%, 0.460	Fixed
Depth of invasion (III + IV vs. I + II)	148	2.13(0.77–5.90)	0.146	57.0%, 0.127	Random
Distant metastasis (Yes vs. No)	277	3.54(2.00–6.28)	<0.001	0.0%, 0.870	Fixed

### Publication bias and sensitivity analysis

**FIGURE 4:**
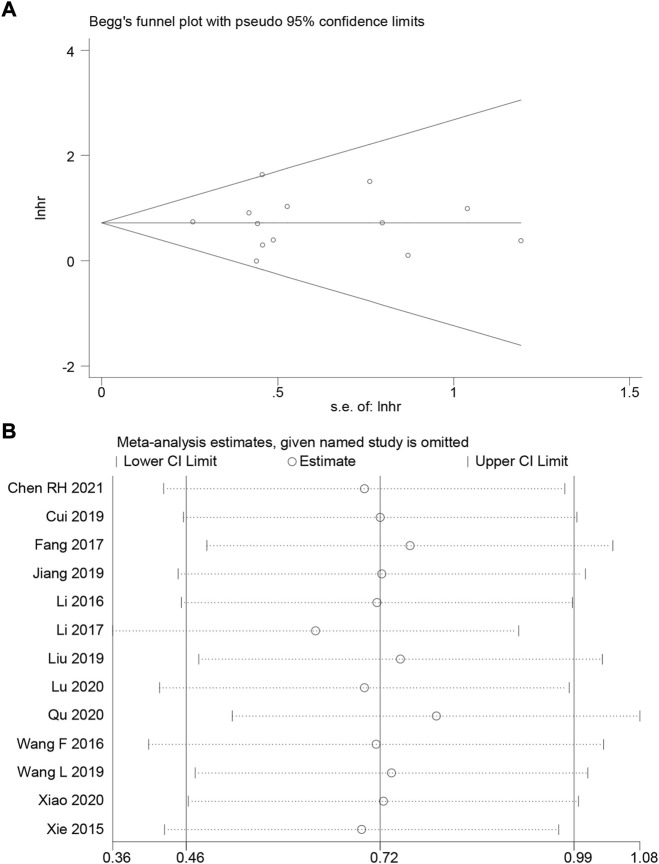
**(A)** Begg’s funnel plot of HOXA-AS2 for overall survival. **(B)** A sensitivity analysis of pooled HR for overall survival.

We employ Begg’s funnel plot and Egger’s regression test to identify publication bias of OS. The shape of Begg’s funnel was essentially symmetrical, with no visible asymmetry (Figure 4A), and Egger’s regression analysis did not reveal the presence of publication bias (Pr > |t| = 0.971). We ran a sensitivity analysis by eliminating one qualified study to analyze the influence of a single study on the result. According to the analysis, the results were not significantly influenced (Figure 4B). This verifies the reliability of the meta-analysis conclusions.

### Validation of HOXA-AS2 expression in public databases

**FIGURE 5:**
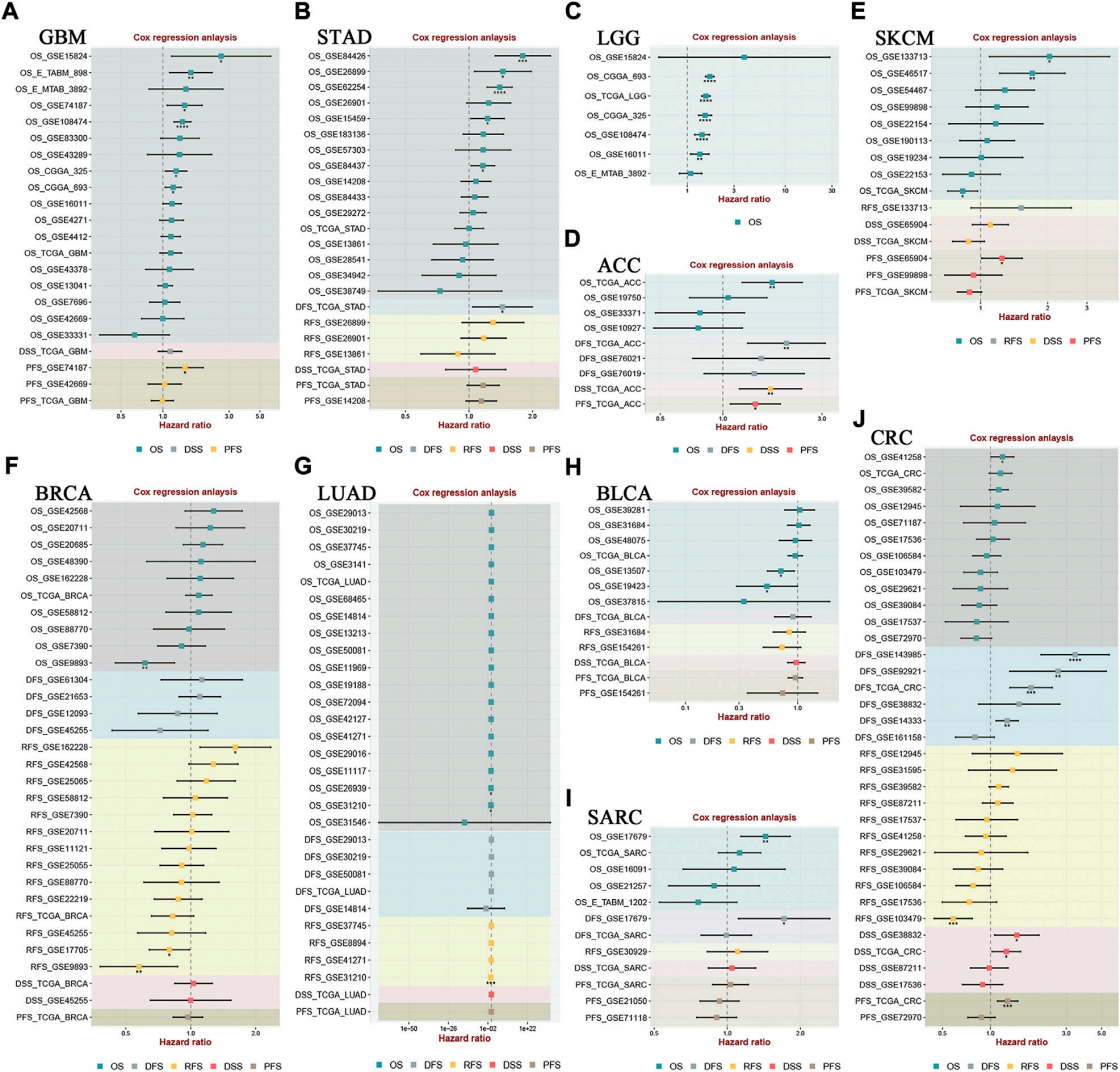
The correlation between HOXA-AS2 expression and survival in different tumor types was analyzed in BEST. Cox regression analysis of **(A)** GBM, **(B)** STAD, **(C)** LGG, **(D)** ACC, **(E)** SKCM, **(F)** BRCA, **(G)** LUAD, **(H)** BLCA, **(I)** SARC, **(J)** CRC. *p < 0.05, **p < 0.01, and ***p < 0.001.

We used the TCGA dataset to analyze the degree of HOXA AS2 expression in various tumors to further corroborate our results. HOXA-AS2 was aberrantly expressed in glioblastoma multiforme (GMB), acute myeloid leukemia (LAML), pancreatic adenocarcinoma (PAAD), and thymoma (THYM), compared to normal controls (Supplementary Figure S1A). A violin plot revealed that the degree of HOXA-AS2 expression in human cancer was highly related to the clinical stage (Supplementary Figure S1B). We used GEPIA to create survival graphs by combining HOXA-AS2 expression data with the OS data of patients with malignancies in the entire TCGA dataset, which included 9491patients separated into high (4741) and low (4750) groups of HOXA-AS2 expression based on median levels. The results showed that increased HOXA-AS2 expression predicted poor OS, confirming the meta-analysis results (Supplementary Figure S1C). Additionally, we explored the link of HOXA-AS2 expression and tumor prognosis using Cox regression model through the BEST online tool. The findings revealed that there was a significant correlation between HOXA-AS2 expression and the prognosis of GBM, stomach adenocarcinoma (STAD), LGG, adrenocortical carcinoma (ACC), skin cutaneous melanoma (SKCM), breast invasive carcinoma (BRCA), lung adenocarcinoma (LUAD), bladder urothelial carcinoma (BLCA), sarcoma (SARC), and CRC in at least two datasets (Figure 5).

### HOXA-AS2 and drug response

**FIGURE 6:**
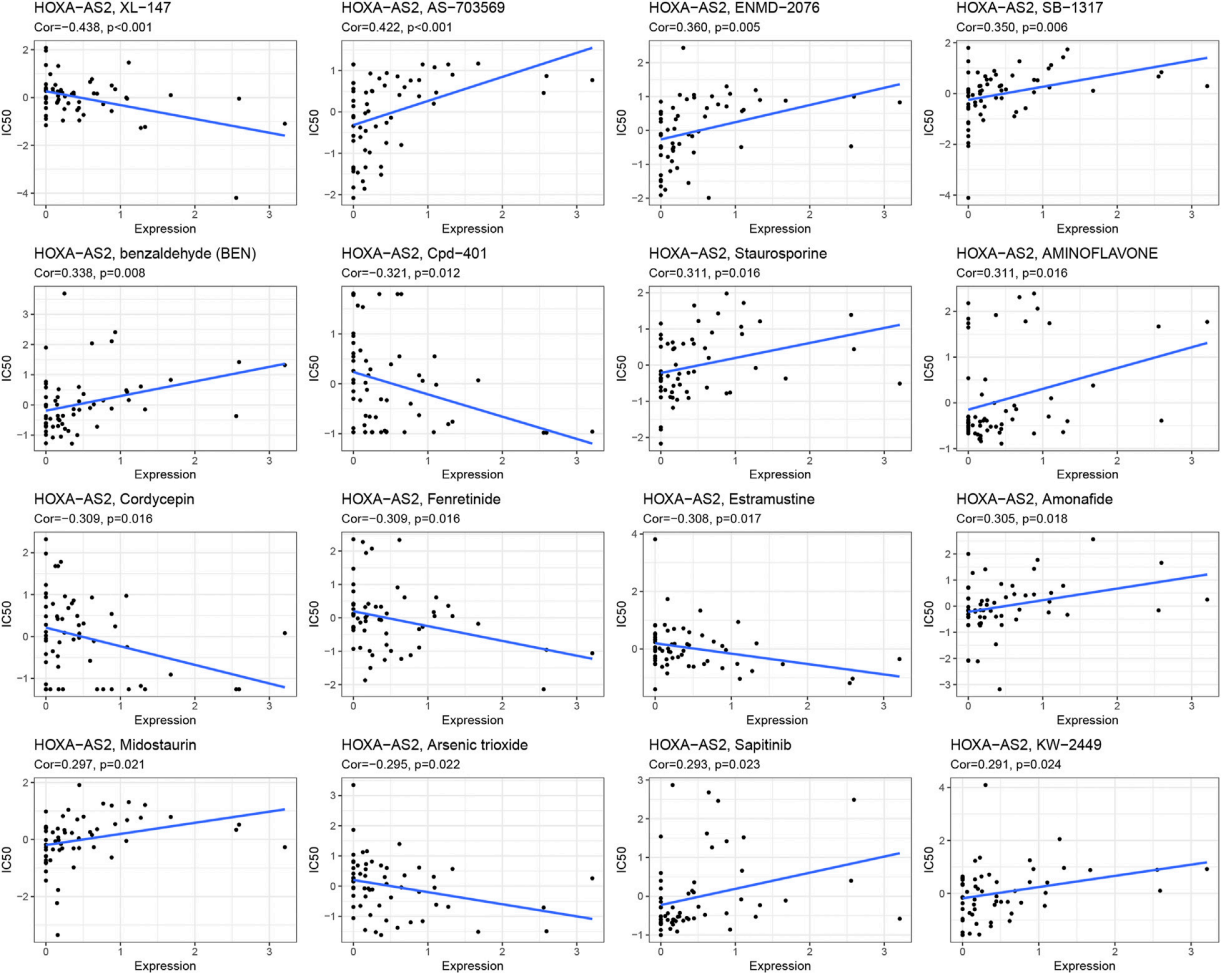
An illustration of the association between HOXA-AS2 expression and expected medication response.

To further explore the significance of HOXA-AS2 in guiding cancer treatment, we analyzed the relationship between HOXA-AS2 and drug response. The results revealed that patients with high HOXA-AS2 expression had a better drug response to XL−147, Cpd−401, cordycepin, fenretinide, estramustine, and arsenic trioxide. In contrast, AS−703569, ENMD−2076, SB−1317, benzaldehyde (BEN), staurosporine, aminoflavone, amonafide, midostaurin, sapitinib, and KW−2449 had a better drug response in patients with low HOXA-AS2 expression (Figure 6).

### Correlation analysis of HOXA-AS2 expression with tumor immunity

**FIGURE 7:**
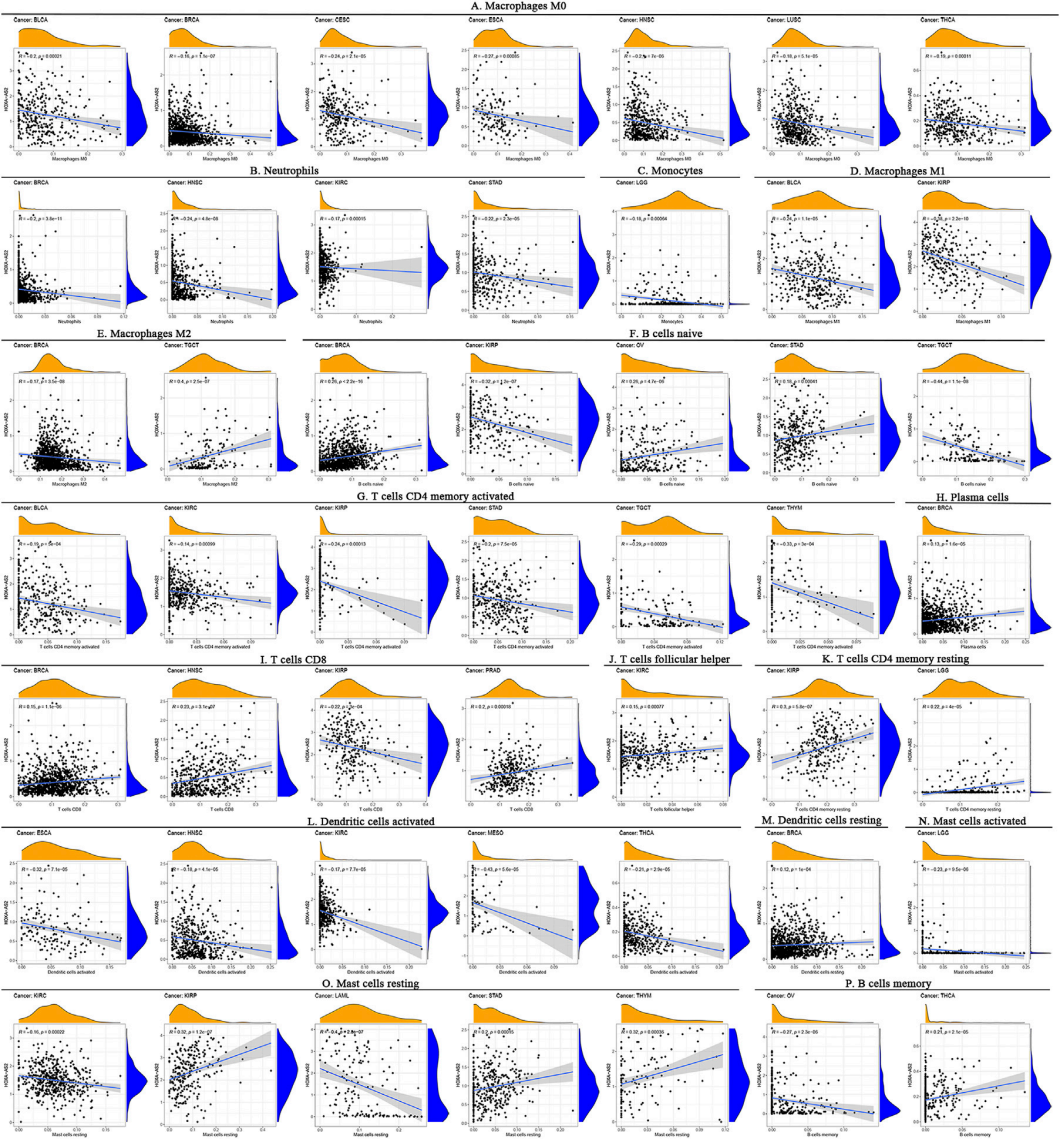
Correlation between HOXA-AS2 gene expression and the level of immune cell infiltration in pan-cancerous tissues. HOXA-AS2 expression significantly correlated with infiltrating levels of M0 macrophages in BLCA, BRCA, CESC, ESCA, NHSC, LUSC, and THCA **(A)**, neutrophils in BRCA, HNSC, KIRC, and STAD **(B)**, monocytes in LGG **(C)**, M1 macrophages in BLCA and KIRP **(D)**, M2 macrophages in BRCA and TGCT **(E)**, naive B cells in BRCA, KIRP, OV, STAD, and TGCT **(F)**, CD4 memory T cells in BLCA, KIRC, KIRP, STAD, TGCT, and THYM **(G)**, plasma cells in BRCA **(H)**, CD8 T cells in BRCA, HNSC, KIRP, and PRAD **(I)**, follicular helper T cells in KIRC **(J)**, resting CD4 memory T cells in KIRP and LGG **(K)**, activated dendritic cells in ESCA, HNSC, KIRC, MESO, and THCA **(L)**, resting DCs in BRCA **(M)**, activated mast cells in LGG **(N)**, resting mast cells in KIRC, KIRP, LAML, STAD, and THYM **(O)**, memory B cells in OV and THCA **(P)**.

Correlation analysis between HOXA-AS2 expression and ICI levels identified remarkable correlations between HOXA-AS2 expression and ICI levels in BRCA (*n* = 7), kidney renal papillary cell carcinoma (KIRP) (*n* = 6), kidney renal clear cell carcinoma (KIRC) (n = 5), head and Neck squamous cell carcinoma (HNSC) (*n* = 4), STAD (*n* = 4), thyroid carcinoma (THCA) (*n* = 3), testicular germ cell tumors (TGCT) (n = 3), BLCA (*n* = 3), LGG (n = 3), THYM (n = 2), ovarian serous cystadenocarcinoma (OV) (*n* = 2), esophageal carcinoma (ESCA) (n = 2), prostate adenocarcinoma (PRAD) (*n* = 1), mesothelioma (MESO) (*n* = 1), lung squamous cell carcinoma (LUSC) (n = 1), LAML (n = 1), and cervical squamous cell carcinoma and endocervical adenocarcinoma (CESC) (n=1). Detailed information on the subpopulations of infiltrating immune cells in various cancer types is illustrated in Figure 7. HOXA-AS2 expression was negatively correlated with the levels of infiltrating M0 macrophages in ESCA, NHSC, CESC, THCA, BRCA, LUSC, and BLCA (Figure 7A). Similarly, HOXA-AS2 expression was negatively associated with the levels of infiltrating neutrophils in STAD, HNSC, KIRC, and BRCA (Figure 7B). In regard to monocytes, their infiltration levels were negatively correlated with the HOXA-AS2 expression in LGG (Figure 7C). HOXA-AS2 expression was also negatively related to the levels of infiltrating M1 macrophages in BLCA, and KIRP (Figure 7D). Moreover, HOXA-AS2 expression was negatively correlated with the levels of infiltrating M2 macrophages in BRCA, but positively associated with TGCT (Figure 7E). HOXA-AS2 expression was positively associated with the levels of infiltrating naive B cells in STAD, OV, and BRCA, but positively associated with KIRP and TGCT (Figure 7F). HOXA-AS2 expression was negatively correlated with the infiltrating levels of activated CD4 memory T cells in BLCA, KIRC, KIRP, STAD, TGCT, and THYM (Figure 7G). Furthermore, HOXA-AS2 expression presented a positive relationship with the levels of infiltrating plasma cells in BRCA (Figure 7H). The levels of infiltrating CD8 T cells were positively associated with HOXA-AS2 expression in PRAD, HNSC, and BRCA, but negatively related in KIRP (Figure 7I). A positive association with infiltrating follicular helper T cells was identified in KIRC (Figure 7J). The levels of infiltrating resting CD4 memory T cells were positively associated with HOXA-AS2 expression in KIRP, and LGG (Figure 7K). HOXA-AS2 expression was negatively associated with the levels of infiltrating activated dendritic cells in MESO, HNSC, KIRC, ESCA, and THCA (Figure 7L). Moreover, HOXA-AS2 expression was positively associated with the levels of infiltrating resting DCs in BRCA (Figure 7M). The levels of infiltrating activated mast cells were negatively associated with HOXA-AS2 expression in LGG (Figure 7N). In contrast, the levels of infiltrating resting mast cells were positively correlated with HOXA-AS2 expression in THYM, STAD, and KIRP, but negatively related in KIRC and LAML (Figure 7O). The levels of infiltrating memory B cells were positively associated with HOXA-AS2 expression in THCA, but negatively associated in OV (Figure 7P).

**FIGURE 8:**
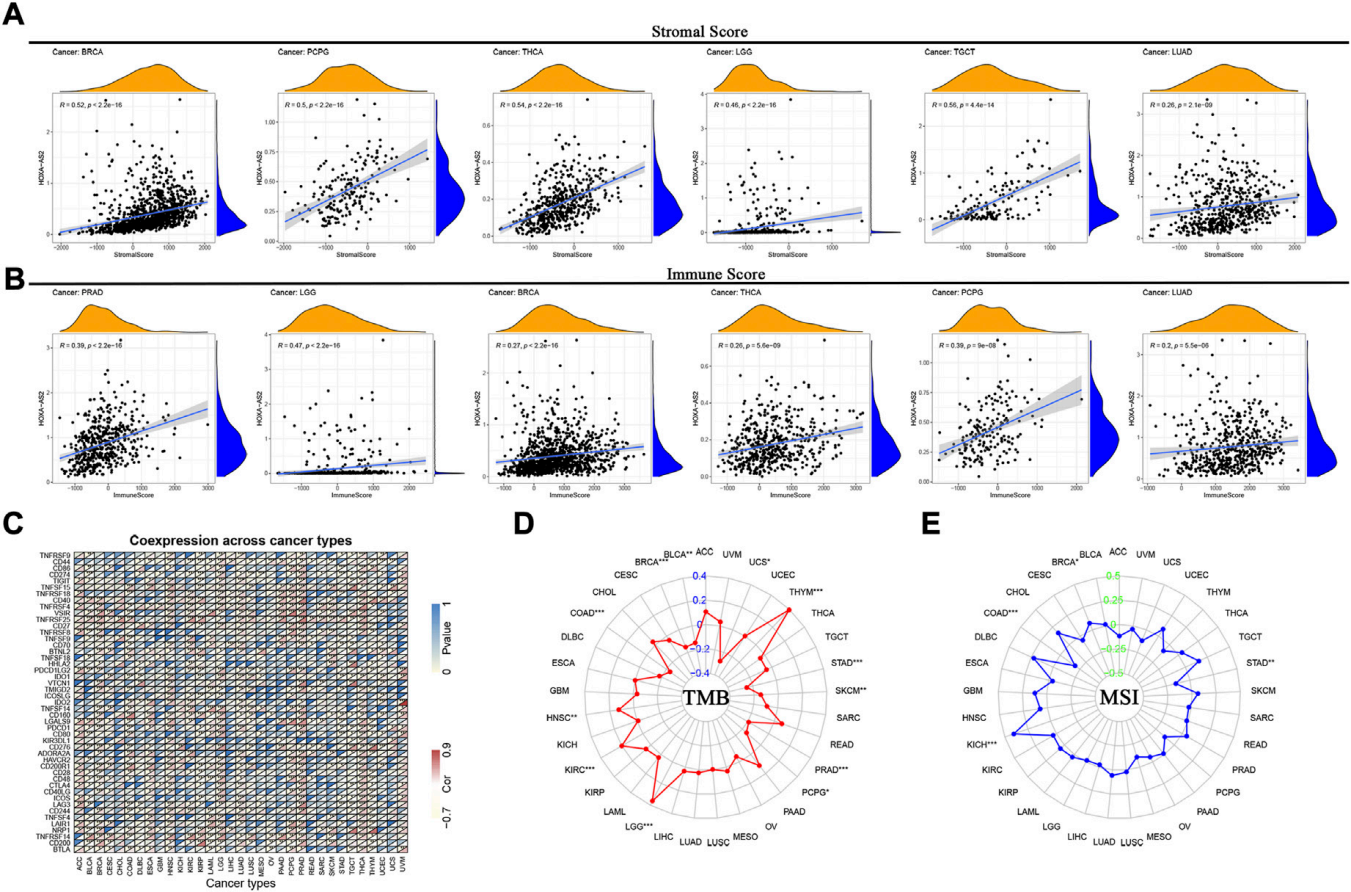
Correlations between HOXA-AS2 expression and immunity, including stromal score, immune score, immune checkpoint genes, TMB and MSI in cancers. **(A)** Correlation of HOXA-AS2 expression with the stromal score in pan-cancer. **(B)** Correlation of HOXA-AS2 expression with the immune score in pan-cancer. **(C)** Correlation of HOXA-AS2 expression and immune checkpoint genes. **(D)** The radar chart displays the correlation of TMB with HOXA-AS2 expression. The red curve indicates the correlation coefficient, and the blue value indicates the range. **(E)** The radar chart displays the correlation of MSI with HOXA-AS2 expression. The blue curve indicates the correlation coefficient, and the green value indicates the range. *p < 0.05, **p < 0.01, and ***p < 0.001.

Furthermore, to explore its relationship with the TME, we analyzed the association of HOXA-AS2 expression with stromal and immune scores. Our findings showed that HOXA-AS2 expression correlated with the stromal scores of 13 cancers, the top 6 tumors were BRCA, pheochromocytoma and paraganglioma (PCPG), THCA, LGG, TGCT, and LUAD (Figure 8A); and with the immune scores of 11 cancers, the top 6 tumors were PRAD, LGG, BRCA, THCA, PCPG, and LUAD (Figure 8B).

Moreover, the correlation of HOXA-AS2 expression with immune checkpoint genes showed that CD44, CD40, VSIR, LGALS9, TNFRSF14 and TNFRSF25 were significantly associated with HOXA-AS2 expression in several cancers, especially in LGG, PRAD, LUAD, THCA, and HNSC (Figure 8C). We evaluated the relation between HOXA-AS2 expression and TMB/MSI as well, and found that there was a significant positive correlation between its expression and TMB in LGG, THYM, KIRC, and HNSC, while there was a significant negative correlation in STAD, BRCA, colon adenocarcinoma (COAD), PRAD, SKCM, BLCA, uterine carcinosarcoma (UCS), and PCPG (Figure 8D). Also, there was a significant positive correlation between HOXA-AS2 expression and MSI in kidney chromophobe (KICH) and BRCA, while there was a significant negative correlation in COAD and STAD (Figure 8E).

### Analysis of HOXA-AS2-related genes

**FIGURE 9:**
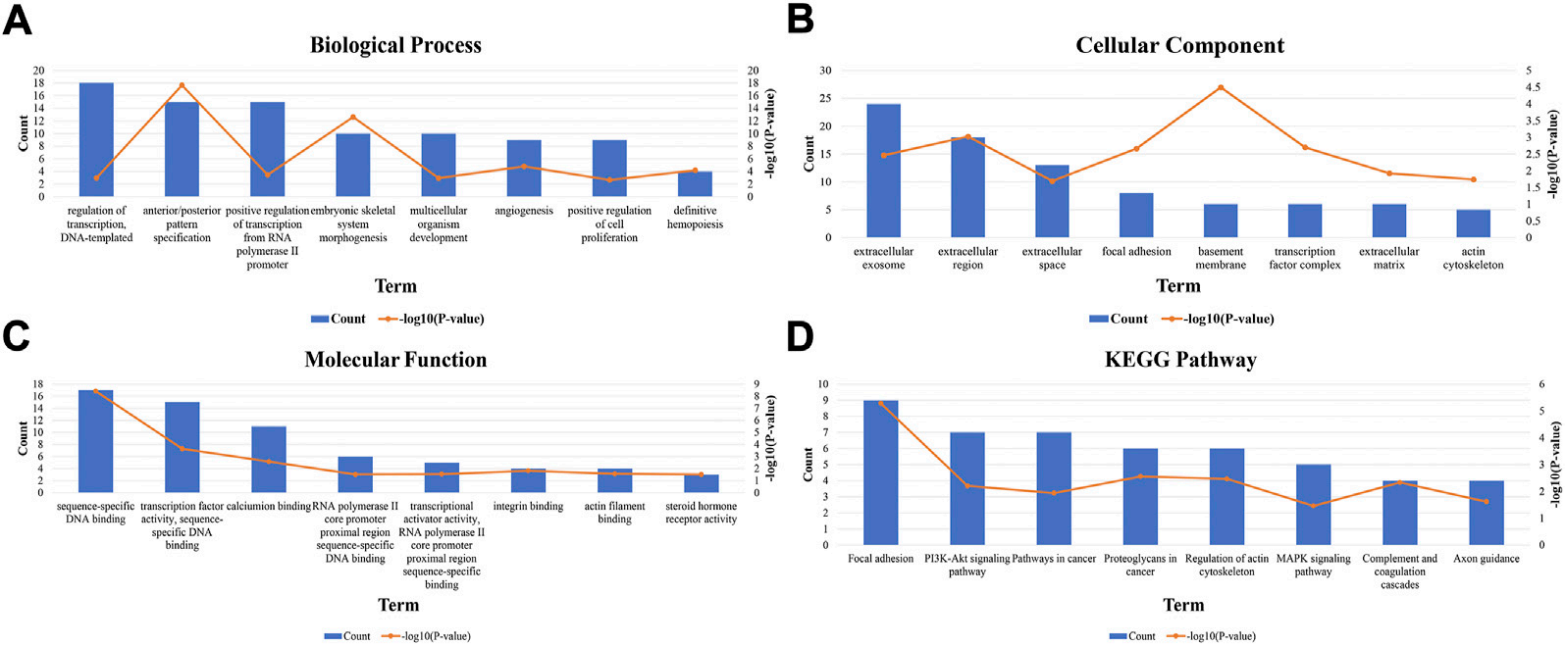
GO terms and the KEGG pathway. **(A)** GO enrichment of target genes in biological process ontolagy (*p* < 0.05).**(B)** GO enichment of target genes in cellular component ontology (*p* < 0.05). **(C)** GO enrichment of target genes in molecular function ontology (*p* < 0.05).**(D)** The top 8 pathways related to the differentially expressed genes by the KEGG database analysis. BP, biological process; CC, cellular component GO, gene ontology analysis; KEGG, Kyoto Encyclopedia of Genes and Genomes; MF, molecular function.

**FIGURE 10:**
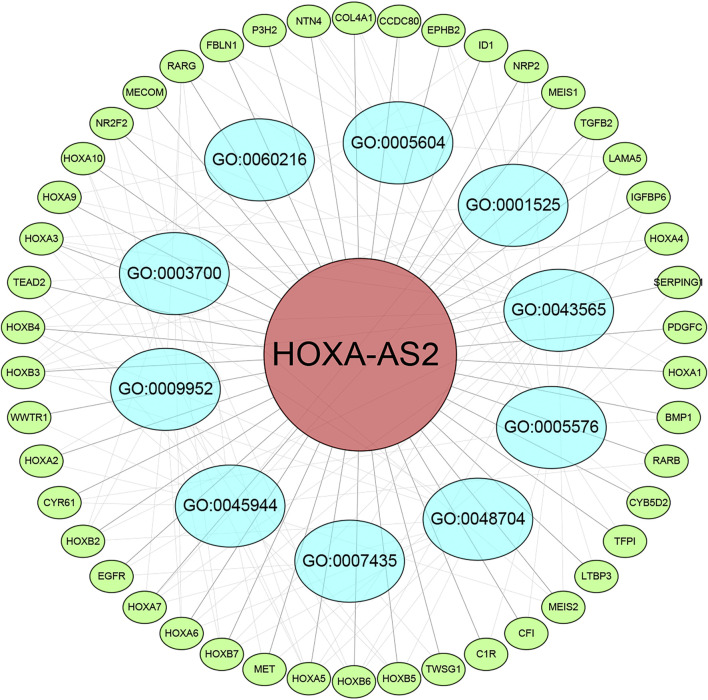
Differentially expressed gene interaction network analysis. Green nodes represent target genes and sky blue nodes represent the related pathway. As indicated in red, HOXA-AS2 localized at the center of the network.

The top 150 genes were screened co-expressed by HOXA-AS2 using the MEM-Multi Experiment Matrix database. HOXA2, HOXA5, and HOXA3 were the top three co-expressed genes ranked by *p*-value and interrelated with HOXA-AS2 expression (Supplementary Figure S2). In addition, GO and KEGG pathway analyses were performed to explore the underlying molecular mechanisms (Figure 9, Table 4), and a signaling pathway network was constructed using Cytoscape software (Figure 10).

**TABLE 4:** Gene ontology analysis of HOXA-AS2-related genes.

GO number	Description	Genes	*P* Value
GO:0009952	anterior/posterior pattern specification	RARG, NR2F2, HOXA10, HOXA9, HOXA3, HOXB4, HOXB3, HOXA2, HOXB2, HOXA7, HOXA6, HOXB7, HOXA5, HOXB6, HOXB5	2.25E-18
GO:0048704	embryonic skeletal system morphogenesis	HOXA3, HOXB4, HOXB3, HOXB2, HOXA7, HOXB7, HOXA6, HOXB6, HOXA5, HOXB5	2.37E-13
GO:0043565	sequence-specific DNA binding	RARG, NR2F2, MEIS2, HOXA10, HOXA9, HOXA3, HOXB4, HOXB3, RARB, HOXB2, HOXA1, HOXA7, HOXA6, HOXB7, HOXA5, HOXB6, HOXA4	3.81E-09
GO:0001525	Angiogenesis	LAMA5, TGFB2, MEIS1, NRP2, ID1, HOXA3, HOXB3, EPHB2, HOXA7	1.56E-05
GO:0005604	basement membrane	LAMA5, CCDC80, COL4A1, NTN4, P3H2, FBLN1	3.18E-05
GO:0060216	definitive hemopoiesis	MEIS1, HOXA9, HOXB4, HOXB3	6.45E-05
GO:0003700	transcription factor activity, sequence-specific DNA binding	RARG, NR2F2, MEIS2, MECOM, ID1, HOXA3, HOXB4, HOXB3, HOXB2, HOXA6, HOXB7, HOXA5, TEAD2, HOXB6, HOXA4	0.000228
GO:0045944	positive regulation of transcription from RNA polymerase II promoter	WWTR1, RARG, MEIS2, CYR61, EGFR, HOXA10, MEIS1, HOXB4, RARB, HOXA2, HOXA7, MET, HOXA5, TEAD2, HOXB5	0.000338
GO:0007435	salivary gland morphogenesis	TGFB2, TWSG1, EGFR	0.000511
GO:0005576	extracellular region	LAMA5, TGFB2, NRP2, C1R, CFI, NTN4, FBLN1, LTBP3, TFPI, CYR61, CYB5D2, BMP1, COL4A1, PDGFC, SERPING1, IGFBP6, EPHB2, MET	0.000943

## Discussion

According to global cancer statistics 2020, there were approximately 19.3 million new cancer cases and 10.0 million cancer deaths worldwide (Sung et al., 2021). Despite the variety of treatments available today, cancer has a high rate of recurrence and death, resulting in increased costs and poor patient prognosis (Huang et al., 2020). For most types of cancer, early detection and treatment improve prognoses. LncRNA was recently found to have huge clinical value in the early diagnosis and novel treatment of patients with cancer (Dunn et al., 2010). Numerous studies showed that lncRNAs were involved in a variety of physiological and pathological processes and had important effects on tumorigenesis and tumor growth (Renganathan and Felley-Bosco, 2017). For instance, prostate cancer-associated transcript 6 (PCAT6) was significantly increased in various cancers. The overexpression of PCAT6 was closely correlated with OS, TNM stage, distant metastasis, LNM, tumor size, and the degree of differentiation in cancer patients (Shi et al., 2021), which may be a new cancer-related biomarker. In recent years, numerous studies found that the HOXA-AS2 was overexpressed in a variety of tumor types, such as CC (Chen and He, 2021), OSCC (Chen et al., 2021), PCa (Xiao and Song, 2020) and so on. The function of HOXA-AS2 in many malignancies has yet to be realized. Thus, we conducted the current meta-analysis to assess the predictive and clinical importance of HOXA-AS2 aberrant expression in cancer patients.

Our meta-analysis found that HOXA-AS2 overexpression was related to a lower chance of survival. We also examined the connection between HOXA-AS2 overexpression and several clinicopathological features. HOXA-AS2 overexpression was associated with tumor stage, LNM, tumor size and distant metastasis. However, there were no significant associations with age, gender, differentiation, or depth of invasion. Furthermore, we performed further analysis of the prognostic role of HOXA-AS2 in various cancers using several public databases. Among them, Cox regression analysis indicated that HOXA-AS2 had a better prognostic value in GBM, STAD, LGG, ACC, SKCM, BRCA, LUAD, BLCA, SARC, and CRC. These results suggest that HOXA-AS2 is a predictor of poor prognosis in cancer patients.

Our findings demonstrated a strong correlation between HOXA-AS2 expression and immunity in multiple tumors. Tumor-infiltrating immune cells play an irreplaceable role in tumor development. Previous research has shown that HOXA-AS2 could influence glioma progression by regulating Treg cell proliferation and immune tolerance (Zhong et al., 2022). We found that HOXA-AS2 was associated with multiple infiltrating immune cells in a variety of tumors. However, the regulatory mechanism of HOXA-AS2 on ICI still remains to be further confirmed by abundant experiments. The interaction between TME and tumor cells is decisive for tumor survival and progression. Immune cells and stromal cells are key components of the TME (Xiao and Yu, 2021). We found that HOXA-AS2 expression correlated with immune cell scores in 11 tumors and with stromal cell scores in 13 tumors. It indicates that HOXA-AS2 has an essential role in the TME. TMB and MSI are well-directed for tumor immunotherapy. Our findings indicated that HOXA-AS2 expression correlated with TMB in 12 cancers and with MSI in 4 cancers. In summary, HOXA-AS2 may further influence the prognosis of cancer patients *via* modulation of tumor immunity.

Although HOXA-AS2 was demonstrated to be a major predictive factor for patients with various malignancies in several studies, the basic principle of how HOXA-AS2 caused cancer remains unknown. According to the results of this study, the overexpression of HOXA-AS2 can significantly aid cancer growth and metastasis. In contrast, inhibited HOXA-AS2 expression, significantly reduced cell proliferation, migration, and invasion, as well as the carcinogenesis process. In PCa, HOXA-AS2 exhibited a negative connection with miR-509–3p. The inhibition of HOXA-AS2 prevented PCa cells from proliferating and migrating (Xiao and Song, 2020). This suggested that HOXA-AS2 could be used as a therapeutic target to treat PCa. In addition, by decreasing miR-520c-3p expression, HOXA-AS2 enhanced the growth and spread of HCC (Wang et al., 2016). HOXA-AS2 was elevated in OSCC tissues and increased OSCC cell proliferation by sponging miR-567/CDK8 (Chen et al., 2021). Table 5 highlights the association between HOXA-AS2 and malignancies to investigate functionally associated genes.

**TABLE 5:** Summary of HOXA-AS2 functional roles and related genes.

Cancer	Expression	Functional role	Related genes	References
Oral squamous cell Carcinoma	Upregulate	Cell proliferation and migration	miR-567/CDK8	Chen et al. (2021)
Colorectal cancer	Upregulate	Cell proliferation and apoptosis	p21 and KLF2	Li et al. (2016)
				Ding et al. (2017)
Breast cancer	Upregulate	Cell proliferation	miR-520c-3p	Fang et al. (2017)
Non-small cell lung cancer	Upregulate	Cell migration, invasion, proliferation, metastasis	miR-520a-3p	Li and Jiang, (2017)
				Cui et al. (2019)
				Liu et al. (2019)
Acute myeloid leukemia	Upregulate	Cell proliferation, invasion	SOX4/PI3K/AKT	Qu et al. (2020)
Hepatocellular Carcinoma	Upregulate	Cell migration, invasion	p-AKT, MMP-2 and MMP-9/miR-520c-3p/GPC3	Wang et al. (2016)
				Zhang et al. (2018)
				Lu et al. (2020)
Bladder cancer	Upregulate	Cell proliferation, invasion	miR-125b/Smad2	Wang et al. (2019a)
Osteosarcoma	Upregulate	Cell migration and invasion	miR-124–3p/E2F3	Wang et al. (2018)
				Wang et al. (2019b)
Glioma	Upregulate	Cell proliferation and invasion and promoted apoptosis	RND3	Xie et al. (2015)
Thyroid	Upregulate	Cell migration and invasion	miR-520c-3p/S100A4	Xia et al. (2018)
				Jiang et al. (2019)
Prostate cancer	Upregulate	Cell proliferation, migration, invasion and EMT	miR-509–3p/PBX3	Xiao and Song, (2020)
Gastric cancer	Upregulate	Cell proliferation and apoptosis	P21/PLK3/DDIT3	Xie et al. (2015)
Cervical cancer	Upregulate	Cell proliferation migration, invasion	miR-509–3p/BTN3A1	Chen and He, (2021)

We used the MEM-Multi Experiment Matrix database to predict target genes and perform the signaling pathway analysis of HOXA-AS2 to further investigate its value. HOXA2, HOXA5, and HOXA3, all of which play important roles in cancer, were strongly associated with HOXA-AS2 expression in our study. Following that, we conducted GO analysis, which indicated that the sequence-specific DNA binding, extracellular exosomes, and angiogenesis of HOXA-AS2 were all significantly related. HOXA AS2 was highly connected to cancer-associated pathways in KEGG analysis.

Nevertheless, this meta-analysis had several limitations. First, some HRs and the corresponding 95% CIs were extracted from KM curves. Second, the qualifying studies were all performed in China, so it is unclear whether the results can be generalized to patients in other countries. To address this limitation, we validated the correlation between HOXA-AS2 expression and prognosis of cancer patients in public databases. Third, the included studies were inconsistent in dividing expression according to cut-off values. Additionally, only a few trials were included, and some cancer types had very low sample sizes. Thus, more clinical investigations should be conducted to assess the potential prognostic role of HOXA-AS2 expression in cancer types that were not included.

## Conclusion

In summary, this meta-analysis found that HOXA-AS2 overexpression was linked to the poor prognosis of cancer patients and could be used as a new prognostic biomarker and therapeutic target for various malignancies. The predictive usefulness of HOXA-AS2 in tumors has to be confirmed in more studies, including other cancer types.

## Data Availability

The original contributions presented in the study are included in the article/Supplementary Materials, further inquiries can be directed to the corresponding authors.

## References

[B1] Bhan A. Soleimani M. Mandal S. S. (2017). Long noncoding RNA and cancer: A new paradigm. Cancer Res. 77 (15), 3965–3981. 10.1158/0008-5472.Can-16-2634 28701486 PMC8330958

[B2] Chen R. He P. (2021). Long noncoding RNA HOXA-AS2 accelerates cervical cancer by the miR-509-3p/BTN3A1 axis. J. Pharm. Pharmacol. 73 (10), 1387–1396. 10.1093/jpp/rgab090 34240204

[B3] Chen R. Wang X. Zhou S. Zeng Z. (2021). LncRNA HOXA-AS2 promotes tumor progression by suppressing miR-567 expression in oral squamous cell carcinoma. Cancer Manag. Res. 13, 5443–5455. 10.2147/cmar.S305946 34267554 PMC8275166

[B4] Chu H. Y. Chen Y. J. Hsu C. J. Liu Y. W. Chiou J. F. Lu L. S. (2020). Physical cues in the microenvironment regulate stemness-dependent homing of breast cancer cells. Cancers (Basel) 12 (8), E2176. 10.3390/cancers12082176 PMC7464848 32764400

[B5] Cui T. J. Lin G. S. Dai Y. M. Zheng J. P. Chen Z. Chen Q. (2019). LncRNA HOXA-AS2 regulates microRNA-216a-5p to promote malignant progression of non-small cell lung cancer. Eur. Rev. Med. Pharmacol. Sci. 23 (3), 264–273. 10.26355/eurrev_201908_18656 31389597

[B6] Ding J. Xie M. Lian Y. Zhu Y. Peng P. Wang J. (2017). Long noncoding RNA HOXA-AS2 represses P21 and KLF2 expression transcription by binding with EZH2, LSD1 in colorectal cancer. Oncogenesis 6 (1), e288. 10.1038/oncsis.2016.84 28112720 PMC5294247

[B7] Dunn B. K. Wagner P. D. Anderson D. Greenwald P. (2010). Molecular markers for early detection. Semin. Oncol. 37 (3), 224–242. 10.1053/j.seminoncol.2010.05.007 20709207

[B8] Fang Y. Wang J. Wu F. Song Y. Zhao S. Zhang Q. (2017). Long non-coding RNA HOXA-AS2 promotes proliferation and invasion of breast cancer by acting as a miR-520c-3p sponge. Oncotarget 8 (28), 46090–46103. 10.18632/oncotarget.17552 28545023 PMC5542252

[B9] Huang S. Yang J. Fong S. Zhao Q. (2020). Artificial intelligence in cancer diagnosis and prognosis: Opportunities and challenges. Cancer Lett. 471, 61–71. 10.1016/j.canlet.2019.12.007 31830558

[B10] Jiang L. Wu Z. Meng X. Chu X. Huang H. Xu C. (2019). LncRNA HOXA-AS2 facilitates tumorigenesis and progression of papillary thyroid cancer by modulating the miR-15a-5p/HOXA3 Axis. Hum. Gene Ther. 30 (5), 618–631. 10.1089/hum.2018.109 30375256

[B11] Kole R. Krainer A. R. Altman S. (2012). RNA therapeutics: Beyond RNA interference and antisense oligonucleotides. Nat. Rev. Drug Discov. 11 (2), 125–140. 10.1038/nrd3625 22262036 PMC4743652

[B12] Li Q. Dai Y. Wang F. Hou S. (2016). Differentially expressed long non-coding RNAs and the prognostic potential in colorectal cancer. Neoplasma 63 (6), 977–983. 10.4149/neo_2016_617 27596298

[B13] Li W. Li J. Mu H. Guo M. Deng H. (2019). MiR-503 suppresses cell proliferation and invasion of gastric cancer by targeting HMGA2 and inactivating WNT signaling pathway. Cancer Cell. Int. 19, 164. 10.1186/s12935-019-0875-1 31249473 PMC6570880

[B14] Li Y. Jiang H. (2017). Up-regulation of long non-coding RNA HOXA-AS2 in non-small cell lung cancer is associated with worse survival outcome. Int. J. Clin. Exp. Pathol. 10 (9), 9690–9696. 31966850 PMC6965976

[B15] Liu Y. Lin X. Zhou S. Zhang P. Shao G. Yang Z. (2019). Long noncoding RNA HOXA-AS2 promotes non-small cell lung cancer progression by regulating miR-520a-3p. Biosci. Rep. 39 (5), BSR20190283. 10.1042/bsr20190283 31064819 PMC6542977

[B16] Lu Q. Gao J. Tang S. Li Z. Wang X. Deng C. (2020). Integrated RNA sequencing and single-cell mass cytometry reveal a novel role of LncRNA HOXA-AS2 in tumorigenesis and stemness of hepatocellular carcinoma. Onco. Targets. Ther. 13, 10901–10916. 10.2147/OTT.S272717 33149607 PMC7602917

[B17] Morlando M. Fatica A. (2018). Alteration of epigenetic regulation by long noncoding RNAs in cancer. Int. J. Mol. Sci. 19 (2), E570. 10.3390/ijms19020570 PMC5855792 29443889

[B18] Qu Y. Wang Y. Wang P. Lin N. Yan X. Li Y. (2020). Overexpression of long noncoding RNA HOXA-AS2 predicts an adverse prognosis and promotes tumorigenesis via SOX4/PI3K/AKT pathway in acute myeloid leukemia. Cell. Biol. Int. 44 (8), 1745–1759. 10.1002/cbin.11370 32369230

[B19] Reinhold W. C. Sunshine M. Liu H. Varma S. Kohn K. W. Morris J. (2012). CellMiner: A web-based suite of genomic and pharmacologic tools to explore transcript and drug patterns in the NCI-60 cell line set. Cancer Res. 72 (14), 3499–3511. 10.1158/0008-5472.Can-12-1370 22802077 PMC3399763

[B20] Renganathan A. Felley-Bosco E. (2017). Long noncoding RNAs in cancer and therapeutic potential. Adv. Exp. Med. Biol. 1008, 199–222. 10.1007/978-981-10-5203-3_7 28815541

[B21] Schmitt A. M. Chang H. Y. (2016). Long noncoding RNAs in cancer pathways. Cancer Cell. 29 (4), 452–463. 10.1016/j.ccell.2016.03.010 27070700 PMC4831138

[B22] Shi S. B. Cheng Q. H. Gong S. Y. Lu T. T. Guo S. F. Song S. M. (2021). PCAT6 may be a new prognostic biomarker in various cancers: A meta-analysis and bioinformatics analysis. Cancer Cell. Int. 21 (1), 370. 10.1186/s12935-021-02079-4 34247605 PMC8273986

[B23] Sung H. Ferlay J. Siegel R. L. Laversanne M. Soerjomataram I. Jemal A. (2021). Global cancer statistics 2020: GLOBOCAN estimates of incidence and mortality worldwide for 36 cancers in 185 countries. Ca. Cancer J. Clin. 71 (3), 209–249. 10.3322/caac.21660 33538338

[B24] Tang L. Chen Y. L. Tang X. Wei D. Xu X. Y. Yan F. (2020). Long noncoding RNA DCST1-AS1 promotes cell proliferation and metastasis in triple-negative breast cancer by forming a positive regulatory loop with miR-873-5p and MYC. J. Cancer 11 (2), 311–323. 10.7150/jca.33982 31897227 PMC6930439

[B25] Wang F. Wu D. Chen J. Chen S. He F. Fu H. (2019a). Long non-coding RNA HOXA-AS2 promotes the migration, invasion and stemness of bladder cancer via regulating miR-125b/Smad2 axis. Exp. Cell. Res. 375 (1), 1–10. 10.1016/j.yexcr.2018.11.005 30412716

[B26] Wang F. Yang H. Deng Z. Su Y. Fang Q. Yin Z. (2016). HOX antisense lincRNA HOXA-AS2 promotes tumorigenesis of hepatocellular carcinoma. Cell. Physiol. biochem. 40 (1-2), 287–296. 10.1159/000452545 27855366

[B27] Wang J. Wang W. Tang Q. Lu L. Luo Z. Li W. (2020a). Long non-coding RNA lnc-GNAT1-1 suppresses liver cancer progression via modulation of epithelial-mesenchymal transition. Front. Genet. 11, 1029. 10.3389/fgene.2020.01029 33193591 PMC7541952

[B28] Wang L. Wang L. Zhang X. (2019b). Knockdown of lncRNA HOXA-AS2 inhibits viability, migration and invasion of osteosarcoma cells by miR-124-3p/E2F3. Onco. Targets. Ther. 12, 10851–10861. 10.2147/ott.S220072 31853184 PMC6914662

[B29] Wang Y. Yang C. Liu X. Zheng J. Zhang F. Wang D. (2020b). Transcription factor AP-4 (TFAP4)-upstream ORF coding 66 aa inhibits the malignant behaviors of glioma cells by suppressing the TFAP4/long noncoding RNA 00520/microRNA-520f-3p feedback loop. Cancer Sci. 111 (3), 891–906. 10.1111/cas.14308 31943575 PMC7060482

[B30] Wang Y. Zhang R. Cheng G. Xu R. Han X. (2018). Long non-coding RNA HOXA-AS2 promotes migration and invasion by acting as a ceRNA of miR-520c-3p in osteosarcoma cells. Cell. Cycle 17 (13), 1637–1648. 10.1080/15384101.2018.1489174 30081707 PMC6133314

[B31] Wu L. Zhu X. Song Z. Chen D. Guo M. Liang J. (2019). Long non-coding RNA HOXA-AS2 enhances the malignant biological behaviors in glioma by epigenetically regulating RND3 expression. Onco. Targets. Ther. 12, 9407–9419. 10.2147/ott.S225678 31819475 PMC6844264

[B32] Xia F. Chen Y. Jiang B. Du X. Peng Y. Wang W. (2018). Long noncoding RNA HOXA-AS2 promotes papillary thyroid cancer progression by regulating miR-520c-3p/S100A4 pathway. Cell. Physiol. biochem. 50 (5), 1659–1672. 10.1159/000494786 30384358

[B33] Xiao S. Song B. (2020). LncRNA HOXA-AS2 promotes the progression of prostate cancer via targeting miR-509-3p/PBX3 axis. Biosci. Rep. 40 (8), BSR20193287. 10.1042/bsr20193287 32519740 PMC7426630

[B34] Xiao Y. Yu D. (2021). Tumor microenvironment as a therapeutic target in cancer. Pharmacol. Ther. 221, 107753. 10.1016/j.pharmthera.2020.107753 33259885 PMC8084948

[B35] Xie M. Sun M. Zhu Y. N. Xia R. Liu Y. W. Ding J. (2015). Long noncoding RNA HOXA-AS2 promotes gastric cancer proliferation by epigenetically silencing P21/PLK3/DDIT3 expression. Oncotarget 6 (32), 33587–33601. 10.18632/oncotarget.5599 26384350 PMC4741787

[B36] Xu X. Zhong Z. Shao Y. Yi Y. (2021). Prognostic value of MEG3 and its correlation with immune infiltrates in gliomas. Front. Genet. 12, 679097. 10.3389/fgene.2021.679097 34220951 PMC8242350

[B37] Ye J. Sun H. Feng Z. Zhang Q. Xia Y. Ji Y. (2019). Prognostic significance of LncRNA GHET1 expression in various cancers: A systematic review and meta-analysis. Biosci. Rep. 39 (10), BSR20190608. 10.1042/bsr20190608 31227613 PMC6822487

[B38] Zhang C. Ren X. He J. Wang W. Tu C. Li Z. (2019). The prognostic value of long noncoding RNA SNHG16 on clinical outcomes in human cancers: A systematic review and meta-analysis. Cancer Cell. Int. 19, 261. 10.1186/s12935-019-0971-2 31632195 PMC6788067

[B39] Zhang Y. Xu J. Zhang S. An J. Zhang J. Huang J. (2018). HOXA-AS2 promotes proliferation and induces epithelial-mesenchymal transition via the miR-520c-3p/GPC3 Axis in hepatocellular carcinoma. Cell. Physiol. biochem. 50 (6), 2124–2138. 10.1159/000495056 30415263

[B40] Zhong C. Tao B. Li X. Xiang W. Peng L. Peng T. (2022). HOXA-AS2 contributes to regulatory T cell proliferation and immune tolerance in glioma through the miR-302a/KDM2A/JAG1 axis. Cell. Death Dis. 13 (2), 160. 10.1038/s41419-021-04471-4 35181676 PMC8857186

[B41] Zhou Q. Hou Z. Zuo S. Zhou X. Feng Y. Sun Y. (2019). LUCAT1 promotes colorectal cancer tumorigenesis by targeting the ribosomal protein L40-MDM2-p53 pathway through binding with UBA52. Cancer Sci. 110 (4), 1194–1207. 10.1111/cas.13951 30690837 PMC6447850

