# Supplementary material for: HOXA-AS2 may be a potential prognostic biomarker in human cancers: A meta-analysis and bioinformatics analysis
Source: Front Genet. 2022 Nov 10;13:944278. doi: 10.3389/fgene.2022.944278 (PMC9686854; doi:10.3389/fgene.2022.944278)
Supplement: Supplementary file 1 [file DataSheet1.pdf]

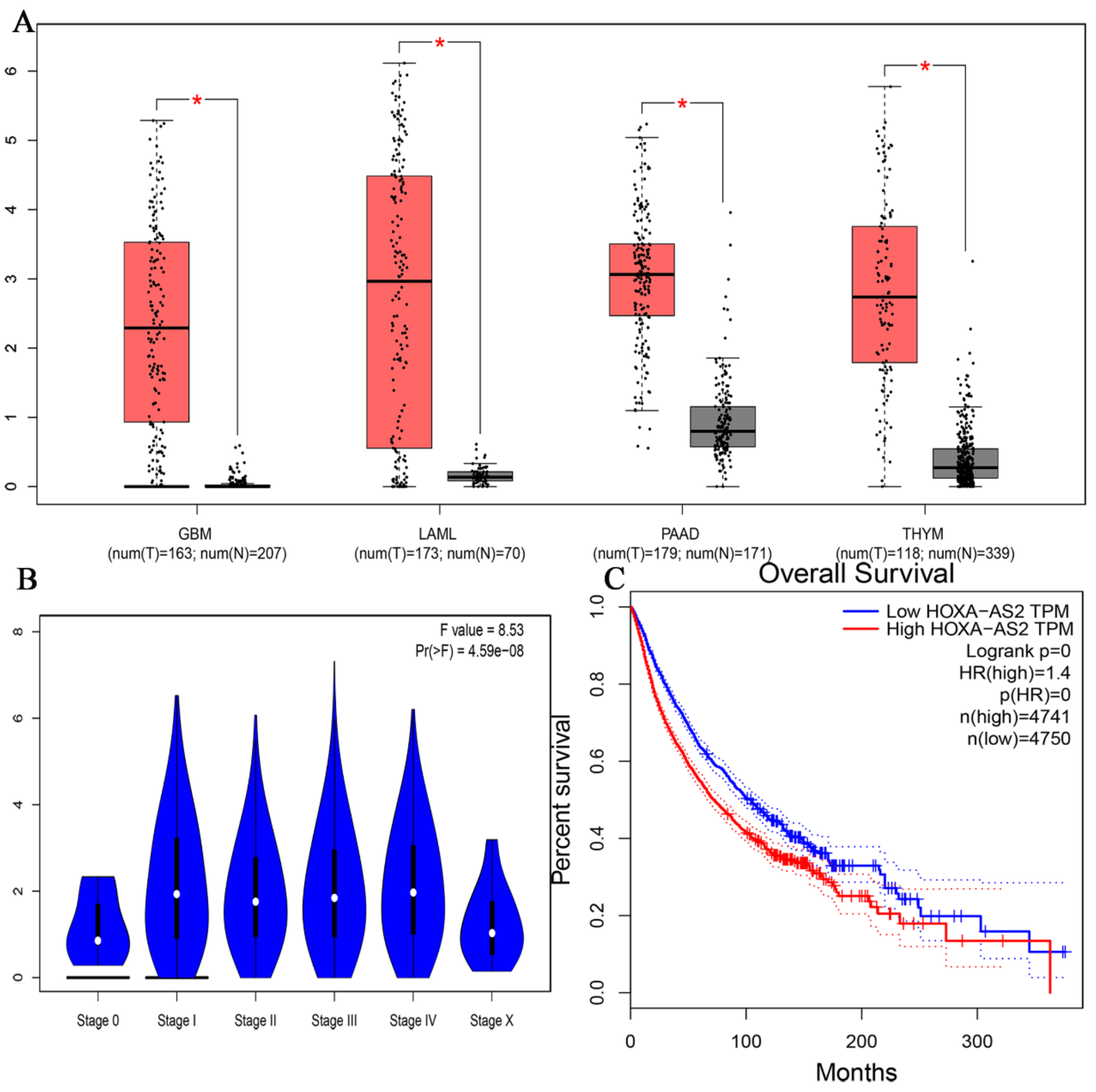

Supplementary Figure S1. Validation of the role of HOXA-AS2 in human cancers in the TCGA dataset. (A) the expression of HOXA-AS2 in cancers (red box) and normal tissues(gray box) ($|Log_2FC| > 1$ and $p < 0.05$), GBM (glioblastoma multiforme), LAML (acute myeloid leukemia), PADD (pancreatic adenocarcinoma) and THYM (thymoma). (B) Violin plot of clinical stage of HOXA-AS2 expression in human pan-cancer; (C) Overall survival plot of HOXA-AS2.

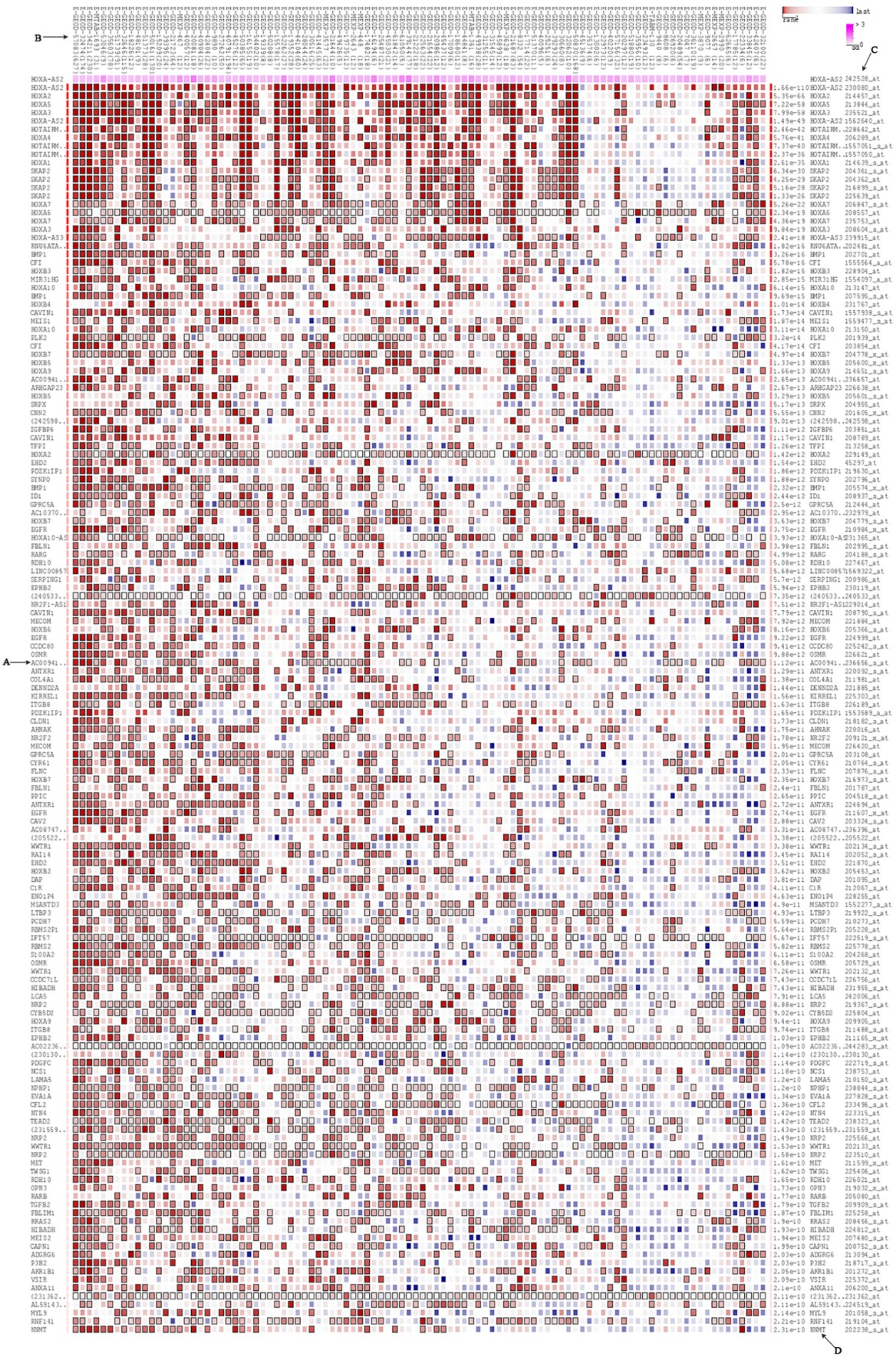

Supplementary Figure S2. The top 150 predicted target genes of HOXA-AS2 by using Multi Experiment Matrix (MEM, http://biit.cs.ut.ee/mem/) website. A, predicted target genes; B, Single experimental data set; C, Gene probes; D, *p* values.
